# Mode of delivery and pregnancy outcomes in preterm birth: a secondary analysis of the WHO Global and Multi-country Surveys

**DOI:** 10.1038/s41598-019-52015-w

**Published:** 2019-10-29

**Authors:** Bao Yen Luong Thanh, Pisake Lumbiganon, Porjai Pattanittum, Malinee Laopaiboon, Joshua P. Vogel, Olufemi T. Oladapo, Cynthia Pileggi-Castro, Rintaro Mori, Kapila Jayaratne, Zahida Qureshi, Joã Souza

**Affiliations:** 1 Department of Epidemiology and Biostatistics, Faculty of Public Health, Khon Kaen University, 123 Moo 16 Mittapap Rd., Nai-Muang, Muang District, Khon Kaen, 40002 Thailand; 2 Department of Biostatistics, Demography and Reproductive Health, Faculty of Public Health, Hue University of Medicine and Pharmacy, Hue University, 06 Ngo Quyen Street, Hue, 530000 Vietnam; 3 Department of Obstetrics and Gynaecology, Faculty of Medicine, Khon Kaen University, 123 Moo 16 Mittapap Rd., Nai-Muang, Muang District, Khon Kaen, 40002 Thailand; 4 UNDP/UNFPA/UNICEF/WHO/World Bank Special Programme of Research, Development and Research Training in Human Reproduction (HRP), Department of Reproductive Health and Research World Health Organization, Avenue Appia 20, Geneva, Switzerland; 5 Independent Consultant, Geneva, Switzerland; 6 Department of Health Policy, National Center for Child Health and Development, 2-10-1 Okura, Setagaya-ku, Tokyo, 157-8535 Japan; 7 Maternal & Child Morbidity & Mortality Surveillance, Family Health Bureau - Ministry of Health, 231 De Saram Place, Colombo 10, Colombo, Sri Lanka; 8 Department of Obstetrics and Gynaecology, School of Medicine, College of Health Sciences, University of Nairobi, Nairobi, Kenya; 9 Department of Social Medicine, University of Sao Paulo, Sao Paulo, Brazil

**Keywords:** Epidemiology, Outcomes research

## Abstract

Many studies have been conducted to examine whether Caesarean Section (CS) or vaginal birth (VB) was optimal for better maternal and neonatal outcomes in preterm births. However, findings remain unclear. Therefore, this secondary analysis of World Health Organization Global Survey (GS) and Multi-country Survey (MCS) databases was conducted to investigate outcomes of preterm birth by mode of delivery. Our sample were women with singleton neonates (15,471 of 237 facilities from 21 countries in GS; and 15,053 of 239 facilities from 21 countries in MCS) delivered between 22 and <37 weeks of gestation. We assessed association between mode of delivery and pregnancy outcomes in singleton preterm births by multilevel logistic regression adjusted for hierarchical data. The prevalences of women with preterm birth delivered by CS were 31.0% and 36.7% in GS and MCS, respectively. Compared with VB, CS was associated with significantly increased odds of maternal intensive care unit admission, maternal near miss, and neonatal intensive care unit admission but significantly decreased odds of fresh stillbirth, and perinatal death. However, since the information on justification for mode of delivery (MOD) were not available, our results of the potential benefits and harms of CS should be carefully considered when deciding MOD in preterm births.

## Introduction

Preterm birth defined as birth before 37 completed weeks of gestation or fewer than 259 days since the first day of a woman’s last menstrual period^1^. A global report in 2015 indicated that preterm birth complications accounted for 17.8% (uncertainty range 15.4 to 19.0%) of all deaths in children under five years old^2^. Due to immature organ systems, newborns who survive are prone to develop both short- and long-term complications, i.e. neurodevelopmental disability, respiratory illnesses, chronic disease in adulthood compared to children born at term^3^. Preterm birth rates appear to increase in many countries. Data from 65 developed countries, Latin America and Caribbean regions showed that its rates has risen from 7.5% in 1990 to 8.6% in 2010^4^.

Many studies have been conducted to assess whether Caesearean Section (CS) or vaginal birth (VB) confers benefits to preterm newborn with minimal harms to mother in preterm birth, but mostly in high-income contries and limited in low- and middle-income countries. However, findings remains unclear. Some observational studies showed that CS could improve outcomes of preterm neonates^5–7^, while other studies suggested that VB was protective against neonatal death^8^. Yet others demonstrated that there was no significant difference in neonatal mortality between both groups^9–14^. Moreover, impact of mode of delivery (MOD) may vary by fetal presentation. For non-vertex-presenting fetuses, CS has been reported to reduce risk of neonatal mortality^15–18^, but evidence was less clear for vertex-presenting fetuses^15,17,19–22^. For mothers, CS seems to cause more severe adverse outcomes^13,23^. Randomized controlled trials (RCTs) had also been conducted to address this question, but planned sample sizes could not be met due to difficulties in recruiting pregnant women^24–28^. A systematic review that assess effects of planned immediate CS versus planned VB for women in preterm labour could include only six RCTs, and involved a total of only 122 women^9^. Consequently, there is little high-quality trial data regarding effects of MOD on outcomes for preterm neonates to guide clinical practice. The World Health Organization (WHO) has indicated that there is insufficient evidence to inform which MOD is optimal for preterm infants^29^.

A clear understanding of maternal and newborn outcomes for different modes of preterm birth could guide clinicians and mothers in making appropriate decisions. This analysis aimed to investigate the relationship between MOD and pregnancy outcomes among women giving preterm birth in two large WHO multi-country surveys.

## Results

**Figure 1:**
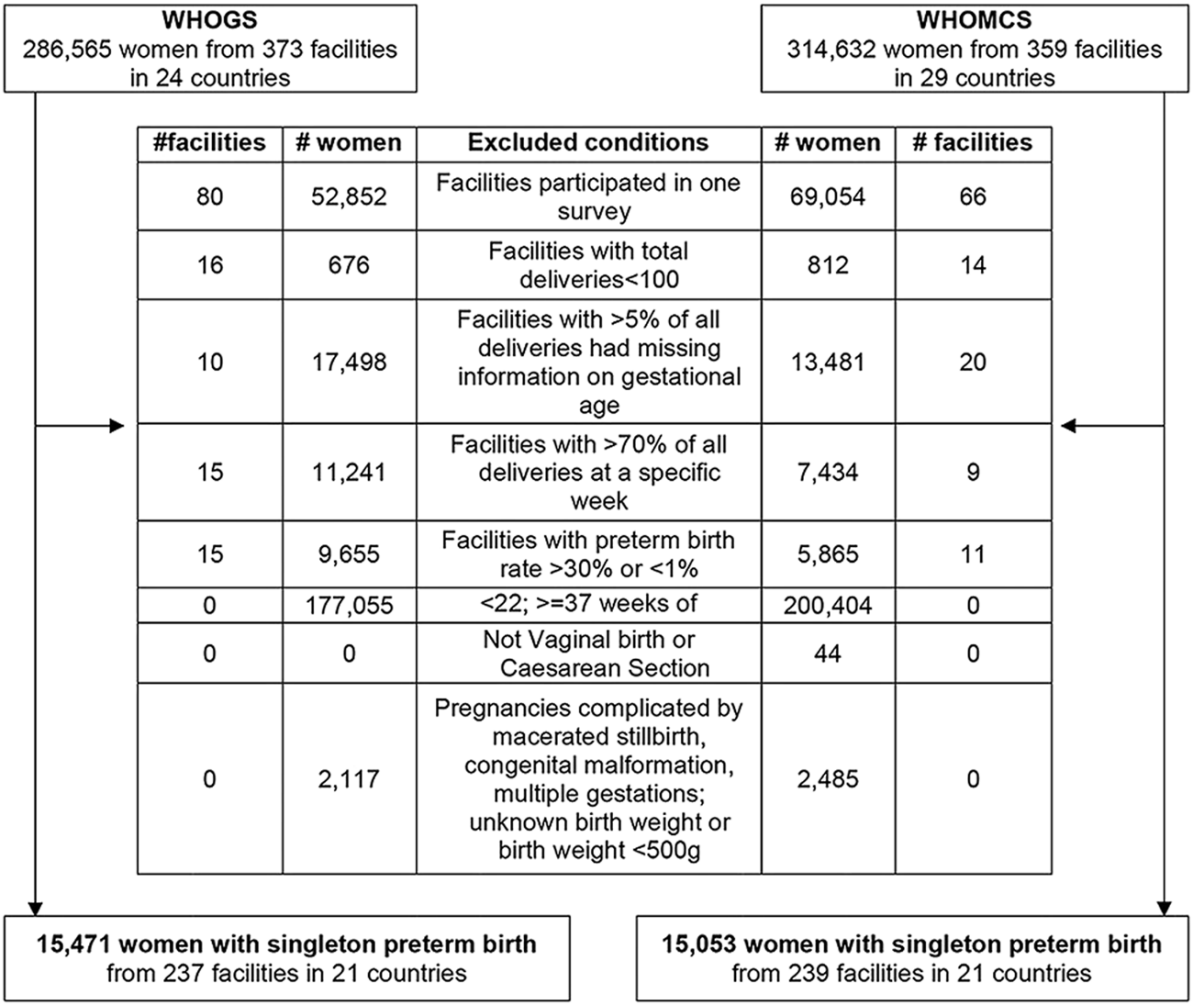
Study population.

A total of 286,565 and 314,623 pregnant women were available from the WHOGS and WHOMCS datasets, respectively. From these two databases, 15,471 and 15,053 women with a singleton delivery from 22 to <37 weeks of gestation were eligible and included in the analysis (Fig. 1). Of the eligible women, the prevalence of CS in these preterm births in WHOGS and WHOMCS were 31.0% and 36.7%, respectively.

Table 1 presented the maternal and perinatal characteristics of the CS and VB groups in WHOGS and WHOMCS. We found that maternal age, maternal education attainment, parity, underlying disease, preeclampsia and eclampsia, gestational age, fetal presentation, corticosteroids administration, newborn’s sex and birthweight were significantly different between CS and VB in both WHOGS and WHOMCS; but marital status was significantly different only in WHOMCS.

**Table 1:** Characteristics of mothers, fetuses and neonates by mode of delivery. *Number of mothers/neonates for each characteristic were not the same due to missing data; NA: Data was not available.

	GS (N = 15,471)				p value	MCS (N = 15,053)				p value
	VB (N = 10,669) n/N(%)		CS (N = 4,802) n/N (%)			VB (N = 9,524) n/N (%)		CS (N = 5,529) n/N (%)		
Maternal characteristics*										
**Marital status**										
Single	1,408/10,629	(13.2)	596/4,792	(12.4)	0.17	1,205/9,458	(12.7)	624/5,491	(11.4)	0.013
Married/cohabiting	9,221/10,629	(86.8)	4,196/4,792	(87.6)		8,253/9458	(87.3)	4,867/5,491	(88.6)	
**Maternal age (years)**										
<20	1,785/10,660	(16.7)	481/4,799	(10.0)	<0.001	1,456/9,501	(15.3)	541/5,513	(9.8)	<0.001
20–34	7,962/10,660	(74.7)	3,437/4,799	(71.6)		7,235/9,501	(76.1)	3,941/5,513	(71.5)	
≥35	913/10,660	(8.6)	881/4,799	(18.4)		810/9,501	(8.5)	1,031/5,513	(18.7)	
**Education attainment (years)**										
<=5	2,250/10,005	(22.5)	640/4,506	(14.2)	<0.001	2,045/8,807	(23.2)	696/5,019	(13.9)	<0.001
6–9	3,629/10,005	(36.3)	1,516/4,506	(33.6)		2,985/8,807	(33.9)	1,475/5,019	(29.4)	
10–12	3,123/10,005	(31.2)	1,543/4,506	(34.2)		2,783/8,807	(31.6)	1,693/5,019	(33.7)	
>12	1,003/10,005	(10.0)	807/4,506	(17.9)		994/8,807	(11.3)	1,152/5,019	(23.0)	
**Parity**										
Nulliparous	5,098/10,615	(48.0)	1,899/4,792	(39.6)	<0.001	4,739/9,516	(49.8)	2,487/5,521	(45.0)	<0.001
Multiparous	5,517/10,615	(52.0)	2,893/4,792	(60.4)		4,777/9,516	(50.2)	3,034/5,521	(55.0)	
**Underlying disease**										
Yes	699/10,595	(6.6)	488/4,768	(10.2)	<0.001	444/9,524	(4.7)	633/5,526	(11.5)	<0.001
No	9,896/10595	(93.4)	4,280/4,768	(89.8)		9,080/9,524	(95.3)	4,893/5,526	(88.5)	
**Preeclampsia**										
Yes	323/10,653	(3.0)	758/4,788	(15.8)	<0.001	350/9,524	(3.7)	933/5,527	(16.9)	<0.001
No	10,330/10,653	(97.0)	4,030/4,788	(84.2)		9,174/9524	(96.3)	4,594/5,527	(83.1)	
**Eclampsia**										
Yes	93/10,652	(0.9)	107/4,788	(2.2)	<0.001	83/9,524	(0.9)	129/5,527	(2.3)	<0.001
No	10,559/10,652	(99.1)	4,681/4,788	(97.8)		9,441/9,524	(99.1)	5,398/5527	(97.7)	
**Perinatal characteristics**										
**Gestational age**										
Extremely preterm	419/10,669	(3.9)	113/4,802	(2.4)	<0.001	480/9,524	(5.0)	135/5,529	(2.4)	<0.001
Very preterm	1,052/10,669	(9.9)	532/4,802	(11.1)		1,191/9,524	(12.5)	653/5,529	(11.8)	
Moderate preterm	9,198/10,669	(86.2)	4,157/4,802	(86.6)		7,853/9,524	(82.5)	4,714/5,529	(85.7)	
Fetal presentation*										
Vertex	10,117/10,664	(94.9)	3,887/4,784	(81.2)	<0.001	8,990/9,513	(94.5)	4,581/5,510	(83.1)	<0.001
Non-vertex	547/10,664	(5.1)	897/4,784	(18.8)		523/9,513	(5.5)	929/5,510	(16.9)	
Corticosteroids*										
No		NA	NA			6,562/9,308	(70.5)	3,172/5,284	(60.0)	<0.001
Yes		NA	NA			2,746/9,308	(29.5)	2,112/5,284	(40.0)	
Sex*										
Female	5,199/10,661	(48.8)	2,222/4,800	(46.3)	0.005	4,644/9,514	(48.8)	2,580/5,522	(46.7)	0.014
Male	5,462/10,661	(51.2)	2,578/4,800	(53.7)		4,870/9,514	(51.2)	2,939/5,522	(53.3)	
**Birth weight**										
≤1,000 g	433/10,669	(4.1)	170/4,802	(3.5)	<0.001	654/9,524	(6.9)	226/5,529	(4.1)	<0.001
>1,000–1,500 g	757/10,669	(7.1)	447/4,802	(9.3)		982/9,524	(10.3)	681/5,529	(12.3)	
>1,500–2,500 g	4,536/10,669	(42.5)	2,210/4,802	(46.0)		4,780/9,524	(50.2)	2,878/5,529	(52.1)	
>2500 g	4,943/10,669	(46.3)	1,975/4,802	(41.1)		3,108/9,524	(32.6)	1,744/5,529	(31.5)	

### Adverse pregnancy outcomes by modes of delivery

For women with a singleton preterm birth, the prevalence of MICU admission were 3.7% in GS and 1.9% in MCS, maternal near miss was 1.7% in MCS (no information on maternal near miss in GS) and maternal death were 0.2% in GS and 0.3% in MCS. Women with CS was significantly associated with increased odds of MICU admission (aORs (95% CIs): 3.7 (1.6–8.5) in GS and 5.0 (3.3–7.7) in MCS), and maternal near miss (aOR (95% CI): 3.7 (2.6–5.4) in MCS). However, it was not significantly different in term of maternal death (aORs (95% CIs): 1.0 (0.4–2.4) in GS and 1.0 (0.5–2.2) in MCS) (Table 2).

**Table 2:** Adverse maternal outcomes by modes of delivery.

Maternal outcomes	GS							MCS						
	n/N (%)			Crude OR (95% CI)		aOR (95% CI)		n/N (%)			Crude OR (95% CI)		aOR (95% CI)	
**MICU admission**	578/	15,464	(3.7)			*		287/	15,035	(1.9)			**‡**	
VB	128/	10,665	(1.2)	1		1		42/	9,518	(0.4)	1		1	
CS	450/	4,799	(9.4)	8.5	(7.0–10.4)	3.7	(1.6–8.5)	245/	5,517	(4.4)	10.5	(7.6–14.6)	5.0	(3.3–7.7)
**Maternal near miss**	NA			NA		NA		257/	15,053	(1.7)			*	
VB	NA			NA		NA		61/	9,524	(0.6)	1		1	
CS	NA			NA		NA		196/	5,529	(3.5)	5.7	(4.3–7.6)	3.7	(2.6–5.4)
**Maternal death**	34/	15,465	(0.2)			**†**		44/	15,050	(0.3)			§	
VB	20/	10,666	(0.2)	1		1		23/	9,524	(0.2)			1	
CS	14/	4,799	(0.3)	1.6	(0.8–3.1)	1.0	(0.4–2.4)	21/	5,529	(0.4)	1.6	(0.9–2.9)	1.0	(0.5–2.2)

*Adjusted for maternal age, maternal education, parity,underlying disease, preeclampsia, eclampsia, mode of delivery, fetal presentation, and FCI; facility was adjusted as a random effect; **†**adjusted for maternal education, underlying disease, preeclampsia, eclampsia, mode of delivery, fetal presentation, and FCI; facility was adjusted as a random effect; **‡** adjusted for maternal age, maternal education, underlying disease, preeclampsia, eclampsia, mode of delivery, fetal presentation, and FCI; facility was adjusted as a random effect; § adjusted for maternal education, parity, underlying disease, preeclampsia, eclampsia, mode of delivery, fetal presentation, and FCI; facility was adjusted as a random effect; || adjusted for maternal education, parity, underlying disease, preeclampsia, eclampsia, mode of delivery, and FCI; facility was adjusted as a random effect.

NA: Data was not available.

The prevalences of Apgar score <7 at 5 minutes, NICU admission, fresh stillbirth, early neonatal and perinatal death were 11.9% and 8.3%, 33.1% and 36.4%, 4.6% and 6.3%, 4.5% and 5.9%, and 8.6% and 11.9%, in GS and MCS, respectively. Delivery by CS was associated with significantly increased odds of NICU admission (aORs (95% CIs): 2.5 (2.1–2.9) in GS and 1.7 (1.4–2.0) in MCS), but decreased odds of fresh stillbirth (aORs (95% CIs): 0.4 (0.2–0.6) in GS and 0.4 (0.3–0.6) in MCS) and perinatal death (aORs (95% CIs): 0.6 (0.5–0.8) in GS and 0.6 (0.5–0.8) in MCS) compared to those delivered by VB. The odds of early neonatal death was not significantly different between CS and VB (aORs (95% CIs): 1.1 (0.8–1.6) in GS and 1.1 (0.9–1.5) in MCS). Neonates delivered by CS was found to be associated with significantly decreased odds of having APGAR score <7 at 5 minutes but only in GS (aOR (95% CI): 0.8 (0.6–0.9)) compared to those delivered by VB (Table 3).

**Table 3:** Adverse perinatal outcomes by modes of delivery.

Perinatal outcomes	GS							MCS						
	n/N (%)			Crude OR (95% CI)		aOR (95% CI)		n/N (%)			Crude OR (95% CI)		aOR (95% CI)	
**APGAR score** <**7 at 5 minutes**	1,833/	15,401	(11.9)			*		1,165/	14,072	(8.3)			‡	
VB	1,299/	10,610	(12.2)	1		1		742/	8,738	(8.5)	1		1	
CS	534/	4,791	(11.1)	0.9	(0.8–1.0)	0.8	(0.6–0.9)	423/	5,334	(7.9)	0.9	(0.8–1.1)	1.1	(0.8–1.4)
**NICU admission**	4,888/	14,746	(33.1)			*		5,133/	14,117	(36.4)			§	
VB	2,640/	10,089	(26.2)	1		1		2,685/	8,774	(30.6)	1		1	
CS	2,248/	4,657	(48.3)	2.6	(2.5–2.8)	2.5	(2.1–2.9)	2,448/	5,343	(45.8)	1.9	(1.8–2.1)	1.7	(1.4–2.0)
**Fresh stillbirth**	719/	15,471	(4.6)			*		954/	15,053	(6.3)			§	
VB	576/	10,669	(5.4)	1		1		766/	9,524	(8.0)	1		1	
CS	143/	4,802	(3.0)	0.5	(0.4–0.7)	0.4	(0.2–0.6)	188/	5.529	(3.4)	0.4	(0.3–0.5)	0.4	(0.3–0.6)
**Early neonatal death**	616/	4,648	(4.5)			**†**		836/	14,061	(5.9)			||	
VB	406/	10,077	(4.0)	1		1		549/	8,739	(6.3)	1		1	
CS	210/	4,648	(4.5)	1.1	(0.9–1.3)	1.1	(0.8–1.6)	287/	5,322	(5.4)	0.9	(0.7–0.9)	1.1	(0.9–1.5)
**Perinatal death**	1,335/	15,444	(8.6)			*		1,790/	15,015	(11.9)			§	
VB	982/	10,653	(9.2)	1		1		1,315/	9,505	(13.8)	1		1	
CS	353/	4,791	(7.4)	0.8	(0.7–0.9)	0.6	(0.5–0.8)	475/	5,510	(8.6)	0.6	(0.5–0.7)	0.6	(0.5–0.8)

*Adjusted for maternal age, maternal education, marital status, parity, underlying disease, preeclampsia, eclampsia, mode of delivery, severity of preterm, fetal presentation, birth weight, sex, and FCI; facility was adjusted as a random effect; **†**adjusted for maternal education, marital status, parity, underlying disease, preeclampsia, eclampsia, mode of delivery, severity of preterm, fetal presentation, birth weight, sex, and FCI; facility was adjusted as a random effect; **‡**adjusted for maternal age, maternal education, marital status, underlying disease, preeclampsia, eclampsia, mode of delivery, severity of preterm, fetal presentation, birth weight, sex, corticosteroids, and FCI; facility was adjusted as a random effect; §adjusted for maternal age, maternal education, marital status, parity, underlying disease, preeclampsia, eclampsia, mode of delivery, severity of preterm, fetal presentation, birth weight, sex, corticosteroids, and FCI; facility was adjusted as a random effect; ||adjusted for maternal age, maternal education, marital status, parity, preeclampsia, eclampsia, mode of delivery, severity of preterm, fetal presentation, birth weight, sex, corticosteroids, and FCI; facility was adjusted as a random effect.

There was a broadly consistent pattern for newborn outcomes, when stratified by vertex and non-vertex presentation. Significant reductions of fresh stillbirth and perinatal death were noticed in non-vertex presenting neonates delivered by CS than those with vertex presenting neonates (Tables S1 and S2).

Similar patterns with the primary analysis of perinatal outcomes were seen across all gestational age subgroups, as well as the regions with consistent reductions in fresh stillbirth associated with CS compared to VB (Tables S3–S7).

## Discussion

Our analysis indicated that in women with preterm singletons, CS was associated with increased odds of MICU admission, maternal near miss (but not maternal death) compared to those delivered by VB. It was also associated with increased odds of NICU admission, but decreased odds of fresh stillbirth and perinatal death. The study also found that the odds of Apgar score <7 at 5 minutes and early neonatal death were not significantly different between CS and VB.

Our major strength was that we utilised two large WHO multi-country databases to evaluate association between MOD and outcomes of preterm birth using standardized data collection methods. This study had a large sample size to detect the association of MOD on relatively rare maternal outcomes (i.e. maternal death and maternal near miss). Although a RCT might be feasible, it would be very difficult to obtain enough sample size to evaluate the association between MOD and important (but statistically rarer) outcomes in preterm birth^24–28^. Therefore, observational designs could be a more practical approach to assess possible associations.

Nonetheless, some limitations need to be considered. First, we had information on mortality, morbidities only up to hospital discharge or seven days after delivery, and no information of long-term pregnancy outcomes, we therefore could not evaluate overall risks and benefits of MOD. However, death and severe morbidities occuring after discharge from hospital should be comparatively rare. Second, despite adjusting for potential confounding factors, there might be some other factors that we could not account for. For example, the decision regarding selection of MOD could be due to specific circumstances, i.e., obstetricians might be unwilling to perform CS for fetuses with little chance of survival in order to avoid any risks to the mothers. Alternatively, a CS might be performed because the fetus had fetal distress. Unfortunately, we didn’t have information about justification for MOD. We also performed subgroup analyses by severity of preterm birth, and excluded those with birth weight <500 g and congenital malformation. The results of subgroup analyses confirmed the primary analysed results. Third, since data were collected from patients’ records, some information might be missing. Fourth, admission criteria to ICU was likely to be different between settings, and women or newborns may not be admitted due to unavailibility of beds. Fifth, data of gestational age was recorded in completed weeks based on the best available obstetric estimate but method of estimation was not recorded. As poor gestational age data can potentially confound our findings, we therefore tried to maximise the validity of this information by excluding facilities with poorer gestational age data. Lastly, our findings can be generalized only to hospitals with more than 1,000 births per year and those able to perform.

Our study found that CS was associated with increased odds of adverse maternal outcomes. The explanation is that CS is an invasive surgical procedure, which puts a mother at higher risks of morbidities and mortality. It is possible that some maternal underlying diseases as well as obstetric complications might lead to CS, and affect maternal outcomes, which complicates the assessment of the risk of CS. However, these factors were already taken into account in our analyses. Like our study, others also illustrated that CS (either for preterm or all births) was associated with increased risks of adverse maternal outcomes^13,30,31^. However, there was no significant difference in the odds of maternal death between CS and VB. This finding was similar to a prospective four-year observational study, involving 3,119 women with singleton preterm pregnacy of 24 to 36 weeks of gestation at 19 academic medical centers in United States^32^.

Among preterm birth, neonates delivered by CS was associated with increased odds of NICU admission. One explanation might be that neonates delivered by CS was due to fetal compromise (i.e. fetal distress). Therefore, they were more likely to be admitted to NICU for further specialized care. Sangkomkamhang *et al*. showed a significantly shorter length of hospital stay in infants delivered vaginally than those delivered by CS^13^.

In vertex-presenting fetuses, MOD was not associated with Apgar score <7 at 5 minutes. This was also found in previous reports^9,13,21^. Likewise, CS was not associated with the odds of early neonatal death. This finding was consistent with previous studies^9,13,21,22,33,34^. However, it was contradicted with another study in United States^20^. This might be due to difference in study population. Werner *et al*. recruited preterm births from 24 to 34 weeks of gestation but only those with appropriate birthweight for gestational age. In vertex presentation, we also found lower odds of fresh stillbirth in CS group than VB group. One explanation could be that CS prevented neonates from a potentially stressful labour encountered in VB. CS also offered vertex-presenting neonates no significant difference for the odds of perinatal death compared to VB. This finding was in line with the work of Wallace *et al*.^27^, a RCT which involved 38 vertex-presenting singleton preterm pregnancies of 26 to 33 weeks of gestation in the United States.

Similar findings were found for moderate preterm birth: CS was associated with decreased odds of perinatal death. Different with our findings, Malloy *et al*. found that CS was associated with increased odds of early neonatal death^8^. The explanation might be that they included a much more restricted group of preterm fetuses with birthweight ranges that varied for specific gestational ages. Different findings were found for very preterm births. The odds of perinatal death was decreased in those delivered by CS in MCS, but was not different in GS. One explanation might be due to improvement of health care services over time, which increased the chance of survival for these neontates. The WHOMCS was conducted more recently (2010–2011) than WHOGS (from 2004–2008) so improvements in quality of care were not unlikely. In MCS, for extremely preterm fetuses, CS was associated with reduced odds of fresh stillbirth, but there were no differences in other outcomes, i.e. Apgar score <7 at 5 minutes, early neonatal death and perinatal death. These findings were consistent with others^15,17^. However, in GS, for extremely preterm birth, CS was protective for fetuses from the odds of perinatal death.

Additionally, our study upheld the hypothesis that for non-vertex fetuses, CS was associated with reduced odds of Apgar score <7 at 5 minutes, fresh stillbirth and perinatal deaths. These might be the results of avoiding difficult labour and delivery which allowed a less stressful or traumatic birth in CS than VB. Previous studies were also in accordance with our findings^15,17,18^. They were retrospective studies of Effer *et al*. (involving 860 singleton live-births at 24 and 25 weeks gestational age in 13 of 17 Canadian tertiary centres)^15^, Lodha *et al*. (3,552 preterm neonates of ≤32 weeks of gestation)^16^, and Reddy *et al*. (768 preterm births of 24 to <32 weeks of gestation)^17^. Another systematic review by Bergenhenegouwen *et al*. also illustrated decreased odds of neonatal death in non-vertex-presenting fetuses delivered by CS^18^.

Our findings suggest that there might be benefit if carrying out CS for preterm birth with non-vertex presentation. For vertex-presenting fetuses, health care providers should counsel pregnant women and their families about benefits and harms before selecting MOD. Further well-designed prospective observational studies are needed to assess the effect of MOD on pregnancy outcomes in preterm births.

## Methods

### Study design and settings

This was a secondary analysis of two facility-based, cross-sectional surveys led by the WHO, the WHO Global Survey (GS) on Maternal and Perinatal Health (2004–2008), and the WHO Multi-Country Survey (MCS) on Maternal and Newborn Health (2010–2011). Details of these surveys have been published elsewhere^35,36^. Briefly, the WHOGS captured data on all women who gave birth in 373 randomly selected health facilities in 24 countries; and the WHOMCS captured data on births and women with severe maternal outcomes in 359 health facilities from 29 countries. For the WHOGS, data collection was conducted from 2004 to 2005 in Africa and Latin America, from 2007 to 2008 in Asia. WHOMCS was conducted from 2010 to 2011 in Africa, Asia, Latin America, and the Middle East. The period of data collection was two or three months depending on the institutional number of annual births. Data for all women (and their babies) were collected from medical records and could not be linked to participants. The technical content of both protocols was reviewed by specialist panels at the UNDP/UNFPA/UNICEF/WHO/World Bank Special Programme of Research, Development and Research Training in Human Reproduction. The Specialist Panel on Epidemiological Research reviewed and approved the WHOGS study protocol for technical content; the Research Project Review Panel (name of panel was changed in 2010) reviewed and approved the technical content of the WHOMCS. The WHOGS and WHOMCS were approved by the WHO Ethical Review Committee and the relevant ethical clearance bodies in participating countries and facilities. Written consent from individual women was not needed because there was no contact between the data collectors (who extracted routine medical record data) and individual women.

### Study population

For this analysis, we included only facilities that participated in both surveys, with at least 100 deliveries in total and with 5% or less missing information on gestational age. We also excluded facilities with unreliable gestational age data, defined as facilities with more than 70% of total deliveries at a specific week of gestational age, or with preterm birth rate more than 30% or less than 1%. Thus, there were 237 and 239 health facilities in WHOGS and WHOMCS, respectively, from 21 countries included in this analysis. We included singleton pregnant women who delivered between 22 and <37 weeks of gestation and their newborns (regardless of vital status at birth). Exclusion criteria included women with ectopic pregnancies or abortion; pregnancies with a documented congenital malformation, or where birth weight was unknown or <500 g (Fig. 1). Pregnancies ending with macerated stillbirth were also excluded, since these likely occurred prior to the onset of labour.

### Definitions of variables

Our main independent variable was MOD, which was categorized as VB or CS. Adverse pregnancy outcomes included adverse maternal and perinatal outcomes. For maternal outcomes, we used maternal intensive care unit (MICU) admission, maternal near miss, and maternal death up to hospital discharge. Maternal near miss was defined as a woman who presented with any life-threatening condition and survived a complication during pregnancy, childbirth or within 7 days of termination of pregnancy (this outcome was captured only for the MCS database). For perinatal outcomes, we used APGAR score <7 at 5 minutes after birth, neonatal intensive care unit (NICU) admission, fresh stillbirth (fetal death, with no signs of maceration), early neonatal death (death of a live born neonate at discharge or within 7 days after birth), and perinatal death (fresh stillbirth or early neonatal death).

Potential confounding variables that were available in WHOGS and WHOMCS databases included both individual and facility characteristics. Individual characteristics were maternal sociodemographic and obstetric characteristics (i.e marital status, maternal age, maternal education, parity); maternal underlying disease (HIV/chronic hypertension/malaria/dengue fever/heart/lung/renal disease/anaemia); obstetric complications (i.e preeclampsia, eclampsia); and fetal and neonatal characteristics (i.e fetal presentation, severity of preterm birth, birth weight, sex). We used the facility complexity index (FCI) to determine the level of services available in each of the facilities and to summarize a facility’s capacity to provide obstetric care (which has been used in previous WHOMCS analyses)^37^. Since there were differences in some composite variables of FCI available in MCS compared to GS, for consistency, we used the FCI score calculated from MCS in both datasets. Scores for the sampled facilities varied from 12 to 57 points.

### Statistical analysis

Characteristics of the participants were described using frequency and percentage for categorical data. To investigate the association between MOD and adverse pregnancy outcomes, odds ratio (OR) and 95% confidence intervals (95% CI) were estimated using generalized linear mixed model with forward stepwise procedure. We also performed sub-group analyses of the risks for adverse perinatal outcomes according to fetal presentation. We expected poorer perinatal outcomes for newborns at earlier gestational ages, hence we also conducted a stratified analysis by severity of preterm birth: extremely preterm birth (<28 weeks), very preterm birth (28- < 32 weeks) and moderately preterm birth (32- < 37 weeks). These models were adjusted for potential confounding factors; facility was also adjusted as a random effect. VB was treated as a reference group. The Akaike’s information criterion^38^ was used to assess the goodness of fit of the model at p < 0.05. All analyses were performed using R program^39^, and the *lme4* package^40^ was used for generalized linear mixed model.

### Ethics approval

The GS and MCS were approved by the WHO Ethical Review Committee and the relevant ethical clearance mechanisms in all facilities.

## Supplementary information

Supplementary Information 

## Data Availability

The datasets generated and/or analyzed during the current study are not publicly available due to they belonged to Department of Reproductive Health and Research, The World Health Organization but could be available from WHO on reasonable request.
