# Supplementary material for: Mode of delivery and pregnancy outcomes in preterm birth: a secondary analysis of the WHO Global and Multi-country Surveys
Source: Sci Rep. 2019 Oct 29;9:15556. doi: 10.1038/s41598-019-52015-w (PMC6820722; doi:10.1038/s41598-019-52015-w)
Supplement: Supplementary file 1 — Supplementary Information [file 41598_2019_52015_MOESM1_ESM.pdf]

Mode of delivery and pregnancy outcomes in preterm birth: a secondary analysis of the WHO Global and Multi-country Surveys

Bao Yen Luong Thanh[1,2], Pisake Lumbiganon[3,*], Porjai Pattanittum[1], Malinee Laopaiboon[1], Joshua P. Vogel[4], Olufemi T. Oladapo[4], Cynthia Pileggi-Castro[5], Rintaro Mori[6], Kapila Jayaratne[7], Zahida Qureshi[8], João Paulo Souza[4,9], on behalf of the WHO Global and Multi-country Surveys on Maternal and Newborn Health Research Network

**Perinatal outcomes**	**GS (N=14,004)**			**MCS (N=13,571)**		
	**n/ N (%)**	**Crude OR (95%CI)**	**aOR (95%CI)**	**n/ N (%)**	**Crude OR (95%CI)**	**aOR (95%CI)**
**APGAR score <7 at 5 minutes**	1,454 / 13,938 (10.4)		*	938 / 12,792 (7.3)		‡
VB	1,060 / 10,060 (10.5)	1	1	624 / 8,363 (7.5)	1	1
CS	394 / 3,878 (10.2)	1.0 (0.8-1.1)	1.0 (0.8-1.3)	314 / 4,429 (7.1)	1.0 (0.8-1.1)	1.2 (0.9-1.6)
**NICU admission**	4,210 / 13,440 (31.3)		*	4,448 / 12,831 (34.7)		§
VB	2,436 / 9,662 (25.2)	1	1	2,463 / 8,393 (29.3)	1	1
CS	1,774 / 3,778 (47.0)	2.6 (2.4-2.8)	2.6 (2.3-3.1)	1,985 / 4,438 (44.7)	1.9 (1.8-2.1)	1.7 (1.5-2.0)
**Fresh stillbirth**	560 / 14,004 (4.0)		*	757 / 13,571 (5.6)		ǁ
VB	451 / 10,117 (4.5)	1	1	613 / 8,990 (6.8)	1	1
CS	109 / 3,887 (2.8)	0.6 (0.5-0.8)	0.5 (0.3-0.9)	144 / 4,581 (3.1)	0.4 (0.3-0.5)	0.5 (0.3-0.7)
**Early neonatal death**	489 / 13,421 (3.6)		†	685 / 12,784 (5.4)		**
VB	343 / 9,651 (3.6)	1	1	463 / 8,361 (5.5)	1	1
CS	146 / 3,770 (3.9)	1.1 (0.9-1.3)	1.3 (0.9-1.8)	222 / 4,423 (5.0)	0.9 (0.8-1.1)	1.3 (0.9-1.6)
**Perinatal death**	1,049 / 13,981 (7.5)		*	1,442 / 13,541 (10.6)		*†
VB	794 / 10,102 (7.9)	1	1	1,076 / 8,974 (12.0)	1	1
CS	255 / 3,879 (6.6)	0.8 (0.7-0.9)	0.7 (0.6-1.0)	366 / 4,567 (8.0)	0.6 (0.5-0.7)	0.9 (0.8-1.1)

Table S1. Adverse perinatal outcomes by different modes of delivery in vertex presenting neonates. *adjusted for maternal age, maternal education, marital status, parity, underlying disease, preeclampsia, eclampsia, mode of delivery, severity of preterm, birth weight, sex, and FCI; facility was adjusted as a random effect; †adjusted for maternal education, marital status, parity, underlying disease, preeclampsia, eclampsia, mode of delivery, severity of preterm, birth weight, sex, and FCI; facility was adjusted as a random effect; ‡adjusted for maternal age, maternal education, marital status, underlying disease, preeclampsia, eclampsia, mode of delivery, severity of preterm, birth weight, sex, corticosteroids, and FCI; facility was adjusted as a random effect; §adjusted for maternal age, maternal education, marital status, parity, underlying disease, preeclampsia, eclampsia, mode of delivery, severity of preterm, birth weight, sex, corticosteroids, and FCI; facility was adjusted as a random effect; ǁadjusted for maternal age, maternal education, marital status, parity, preeclampsia, eclampsia, mode of delivery, severity of preterm, birth weight, corticosteroids and FCI; facility was adjusted as a random effect; **adjusted for maternal age, maternal education, marital status, parity, eclampsia, mode of delivery, severity of preterm, birth weight, sex, corticosteroids, and FCI; facility was adjusted as a random effect; *†adjusted for maternal age, maternal education, marital status, parity, preeclampsia, eclampsia, mode of delivery, severity of preterm, birth weight, sex, corticosteroids and FCI; facility was adjusted as a random effect.

Perinatal outcomes	GS (N=1,444)				MCS (N=1,452)			
	n/ N (%)		Crude OR (95%CI)	aOR (95%CI)	n/ N (%)		Crude OR (95%CI)	aOR (95%CI)
**APGAR score <7 at 5 minutes**	373/ 1,440	(25.9)		*	226 / 1,261	(17.9)		‖
VB	237/ 545	(43.5)	1	1	117 / 373	(31.4)	1	1
CS	136/ 895	(15.2)	0.2 (0.1-0.3)	0.2 (0.1-0.4)	109 / 888	(12.3)	0.3 (0.2-0.4)	0.5 (0.3-0.8)
**NICU admission**	667 / 1,285	(51.9)		†	669 / 1,263	(53.0)		**
VB	203 / 423	(48.0)	1	1	219 / 376	(58.2)	1	1
CS	464 / 862	(53.8)	1.3 (1.0-1.6)	1.6 (1.1-2.3)	450 / 887	(50.7)	0.7 (0.6-0.9)	0.8 (0.6-1.2)
**Fresh stillbirth**	157 / 1,444	(10.9)		‡	190 / 1,452	(13.1)		*†
VB	124 / 547	(22.7)	1	1	147 / 523	(28.1)	1	1
CS	33 / 897	(3.7)	0.1 (0.09-0.2)	0.2 (0.1-0.3)	43 / 929	(13.1)	0.1 (0.09-0.2)	0.1 (0.03-0.3)
**Early neonatal death**	124 / 1,284	(9.7)		§	148 / 1,255	(11.8)		*§
VB	62 / 422	(14.7)	1	1	84 / 374	(22.5)	1	1
CS	62 / 862	(7.2)	0.5 (0.3-0.7)	0.9 (0.5-1.5)	64 / 881	(7.3)	0.3 (0.2-0.4)	0.6 (0.4-0.9)
**Perinatal death**	281 / 1,441	(19.5)		*	338 / 1,445	(23.4)		*‖
VB	186 / 546	(34.1)	1	1	231 / 521	(44.3)	1	1
CS	95 / 895	(10.6)	0.2 (0.1-0.3)	0.4 (0.2-0.6)	107 / 924	(11.6)	0.16 (0.1-0.21)	0.3 (0.2-0.4)

Table S2. Adverse perinatal outcomes by modes of delivery in non-vertex presenting neonates. *adjusted for maternal age, maternal education, parity, underlying disease, eclampsia, mode of delivery, severity of preterm, birth weight, and FCI; facility was adjusted as a random effect; †adjusted for maternal education, marital status, parity, underlying disease, preeclampsia, mode of delivery, severity of preterm, birth weight, and FCI; facility was adjusted as a random effect; ‡adjusted for maternal age, maternal education, marital status, underlying disease, eclampsia, mode of delivery, severity of preterm, birth weight, sex, and FCI; facility was adjusted as a random effect; §adjusted for maternal education, parity, underlying disease, preeclampsia, mode of delivery, severity of preterm, birth weight, and FCI; facility was adjusted as a random effect; ‖adjusted for maternal education, marital status, underlying disease, mode of delivery, severity of preterm, birth weight, corticosteroids, and FCI; facility was adjusted as a random effect; **adjusted for maternal age, maternal education, marital status, preeclampsia, mode of delivery, severity of preterm, birth weight, corticosteroids, and FCI; facility was adjusted as a random effect; *†adjusted for maternal education, marital status, underlying disease, mode of delivery, severity of preterm, birth weight, sex, corticosteroids and FCI; facility was adjusted as a random effect; *‡adjusted for maternal education, marital status, underlying disease, preeclampsia, mode of delivery, severity of preterm, birth weight, sex, corticosteroids and FCI; facility was adjusted as a random effect; *§adjusted for maternal education, mode of delivery, severity of preterm, birth weight, corticosteroids and FCI; facility was adjusted as a random effect; *‖adjusted for maternal education, marital status, underlying disease, preeeclampsia, mode of delivery, severity of preterm, birth weight, sex, corticosteroids and FCI; facility was adjusted as a random effect.

Perinatal outcomes	GS (N=532)			MCS (N=615)		
	n/ N (%)	Crude OR (95%CI)	aOR (95%CI)	n/ N (%)	Crude OR (95%CI)	aOR (95%CI)
**APGAR score <7 at 5 minutes**	341 / 530 (64.3)		*	183 / 389 (47.0)		**
VB	289 / 417 (69.3)	1	1	136 / 276 (49.3)	1	1
CS	52 / 113 (46.0)	0.4 (0.3-0.6)	0.4 (0.2-0.8)	47 / 113 (41.6)	0.7 (0.5-1.1)	0.8 (0.4-1.7)
**NICU admission**	276 / 370 (74.6)		†	301 / 392 (76.8)		*†
VB	195 / 273 (71.4)	1	1	200 / 282 (70.9)	1	1
CS	81 / 97 (83.5)	2.0 (1.1-3.7)	2.0 (0.8-5.5)	101 / 110 (91.8)	4.6 (2.2-9.5)	7.1 (1.8-28.9)
**Fresh stillbirth**	160 / 532 (30.1)		‡	230 / 615 (37.4)		*‡
VB	144 / 419 (34.4)	1	1	207 / 480 (43.1)	1	1
CS	16 / 113 (14.2)	0.3 (0.2-0.6)	0.3 (0.2-0.7)	23 / 135 (17.0)	0.3 (0.2-0.4)	0.3 (0.2-0.6)
**Early neonatal death**	169 / 368 (45.9)		§	195 / 381 (51.2)		*§
VB	129 / 271 (47.6)	1	1	137 / 271 (50.6)	1	1
CS	40 / 97 (41.2)	0.8 (0.5-1.2)	0.9 (0.4-1.8)	58 / 110 (52.7)	1.1 (0.7-1.7)	2.0 (0.9-4.1)
**Perinatal death**	329 / 528 (62.3)		‖	425 / 611 (69.6)		*‖
VB	273 / 415 (65.8)	1	1	344 / 478 (72.0)	1	1
CS	56 / 113 (49.6)	0.5 (0.3-0.8)	0.5 (0.3-1.0)	81 / 133 (60.9)	0.6 (0.4-0.9)	1.2 (0.6-2.4)

Table S3. Adverse perinatal outcomes by modes of delivery in extremely preterm birth (22-<28 weeks). *adjusted for maternal education, marital status, parity, underlying disease, mode of delivery, gestational age, fetal presentation, birth weight, and FCI; facility was adjusted as a random effect; †adjusted for maternal education, marital status, parity, underlying disease, mode of delivery, gestational age, fetal presentation, birth weight, and FCI; facility was adjusted as a random effect; ‡adjusted for maternal education, parity, underlying disease, eclampsia, mode of delivery, gestational age, fetal presentation, birth weight and FCI; facility was adjusted as a random effect; §adjusted for maternal education, marital status, underlying disease, preeclampsia, mode of delivery, gestational age, fetal presentation, birth weight, and FCI; facility was adjusted as a random effect; ‖adjusted for maternal education, parity, underlying disease, preeclampsia, eclampsia, mode of delivery, gestational age, fetal presentation, birth weight and FCI; facility was adjusted as a random effect; **adjusted for maternal education, marital status, mode of delivery, gestational age, fetal presentation, birth weight, corticosteroids and FCI; facility was adjusted as a random effect; *†adjusted for materal age, maternal education, marital status, mode of delivery, gestational age, birth weight, corticosteroids and FCI; facility was adjusted as a random effect; *‡adjusted for maternal education, marital status, mode of delivery, gestational age, fetal presentation, corticosteroids and FCI; facility was adjusted as a random effect; *§adjusted for maternal education, marital status, eclampsia, mode of delivery, gestational age, birth weight,corticosteroids and FCI; facility was adjusted as a random effect; *‖adjusted for maternal age, maternal education, marital status, eclampsia, mode of delivery, gestational age, fetal presentation, birth weight, sex, corticosteroids and FCI; facility was adjusted as a random effect.

Perinatal outcomes	GS (N=1,584)			MCS (N=1,844)		
	n/ N (%)	Crude OR (95%CI)	aOR (95%CI)	n/ N (%)	Crude OR (95%CI)	aOR (95%CI)
**APGAR score <7 at 5 minutes**	506 / 1,580 (32.0)		*	391 / 1,541 (25.4)		‖
VB	360 / 1,049 (34.3)	1	1	244 / 946 (25.8)	1	1
CS	146 / 531 (27.5)	0.7 (0.5-0.9)	0.7 (0.5-0.9)	147 / 595 (24.7)	0.9 (0.8-1.2)	1.1 (0.7-1.8)
**NICU admission**	904 / 1,387 (65.2)		†	1,148 / 1,543 (74.4)		**
VB	528 / 903 (58.5)	1	1	666 / 946 (70.4)	1	1
CS	376 / 484 (77.7)	2.5 (1.9-3.2)	2.1 (1.4-3.1)	482 / 597 (80.7)	1.8 (1.4-2.3)	1.2 (0.8-1.8)
**Fresh stillbirth**	196 / 1,584 (12.4)		‡	309 / 1,844 (16.8)		*†
VB	149 / 1,052 (14.2)	1	1	250 / 1,191 (21.0)	1	1
CS	47 / 532 (8.8)	0.6 (0.4-0.8)	0.6 (0.4-0.9)	59 / 653 (9.0)	0.4 (0.3-0.5)	0.4 (0.2-0.6)
**Early neonatal death**	237 / 1,383 (17.1)		§	321 / 1,527 (21.0)		*‡
VB	144 / 902 (16.0)	1	1	222 / 936 (23.7)	1	1
CS	93 / 481 (19.3)	1.3 (0.9-1.7)	1.3 (0.8-2.0)	99 / 591 (16.8)	0.7 (0.5-0.8)	0.7 (0.5-1.1)
**Perinatal death**	433 / 1,579 (27.4)		‡	630 / 1,836 (34.3)		*§
VB	293 / 1,051 (27.9)	1	1	472 / 1,186 (39.8)	1	1
CS	140 / 528 (26.5)	0.9 (0.7-1.2)	0.8 (0.5-1.2)	158 / 650 (24.3)	0.5 (0.4-0.6)	0.6 (0.4-0.9)

Table S4. Adverse perinatal outcomes by modes of delivery in very preterm birth (28-<32 weeks). *adjusted for maternal age, maternal education, marital status, underlying disease, eclampsia, mode of delivery, gestational age, fetal presentation,birth weight, and FCI; facility was adjusted as a random effect; †adjusted for maternal education, marital status, underlying disease, preeclampsia, mode of delivery, birth weightand FCI; facility was adjusted as a random effect; ‡adjusted for maternal education, marital status, parity, underlying disease, eclampsia, mode of delivery, gestational age, fetal presentation, birth weight, sex and FCI; facility was adjusted as a random effect; §adjusted for maternal education, parity, underlying disease, mode of delivery, gestational age, fetal presentation, birth weight, sex and FCI; facility was adjusted as a random effect; ||adjusted for maternal age, maternal education, marital status, mode of delivery, gestational age, fetal presentation,birth weight, sex, corticosteroids and FCI; facility was adjusted as a random effect; **adjusted for maternal age, maternal education, marital status, mode of delivery, gestational age, fetal presentation, birth weight, corticosteroids and FCI; facility was adjusted as a random effect; *†adjusted for maternal age, maternal education, marital status, mode of delivery, preeclampsia, eclampsia, gestational age, fetal presentation, birth weight, sex, corticosteroids and FCI; facility was adjusted as a random effect;*‡adjusted for maternal age, maternal education, marital status, parity, eclampsia, mode of delivery, gestational age, fetal presentation, birth weight, sex, corticosteroids and FCI; facility was adjusted as a random effect; *§adjusted for maternal age, maternal education, marital status, eclampsia, mode of delivery, gestational age, fetal presentation, birth weight, sex, corticosteroids and FCI; facility was adjusted as a random effect.

Perinatal outcomes	GS (N=13,355) n/ N (%)	GS (N=13,355) Crude OR (95%CI)	GS (N=13,355) aOR (95%CI)	MCS (N=12,594) n/ N (%)	MCS (N=12,594) Crude OR (95%CI)	MCS (N=12,594) aOR (95%CI)
**APGAR score <7 at 5 minutes**	986/ 13,291 (7.4)		*	591/ 12,142 (4.9)		‡
VB	650/ 9,144 (7.1)	1	1	362/ 7,516 (4.8)	1	1
CS	336/ 4,147 (8.1)	1.15 (1.0-1.3)	0.9 (0.6-1.2)	229/ 4,626 (5.0)	1.0 (0.9-1.2)	1.2 (0.9-1.5)
**NICU admission**	3,708/ 12,989 (28.5)		*	3,683/ 12,182 (30.2)		§
VB	1,917/ 8,913 (21.5)	1	1	1,819/ 7,546 (24.1)	1	1
CS	1,791/ 4,076 (43.9)	2.9 (2.6-3.1)	2.7 (2.3-3.2)	1,864/ 4,636 (40.2)	2.1 (2.0-2.3)	1.8 (1.6-2.1)
**Fresh stillbirth**	363/ 13,355 (2.7)		*	415/ 12,594 (3.3)		§
VB	283/ 9,198 (3.1)	1	1	309/ 7,853 (3.9)	1	1
CS	80/ 4,157 (1.9)	0.6 (0.5-0.8)	0.4 (0.2-0.7)	106/ 4,741 (2.2)	0.6 (0.5-0.7)	0.4 (0.3-0.7)
**Early neonatal death**	210/ 12,974 (1.6)		†	320/ 12,153 (2.6)		‖
VB	133/ 8,904 (1.5)	1	1	190/ 7,532 (2.5)	1	1
CS	77/ 4,070 (1.9)	1.3 (0.9-1.7)	1.2 (0.8-1.7)	130/ 4,621 (2.8)	1.1 (0.9-1.4)	0.8 (0.4-1.3)
**Perinatal death**	573/ 13,337 (4.3)		*	735/ 12,568 (5.8)		§
VB	416/ 9,187 (4.5)	1	1	499 / 7,841 (6.4)	1	1
CS	157/ 4,150 (3.8)	0.8 (0.7-1.0)	0.5 (0.4-0.8)	236 / 4,727 (5.0)	0.8 (0.7-0.9)	0.6 (0.5-0.9)

Table S5. Adverse perinatal outcomes by different modes of delivery in moderate preterm birth (32-<37 weeks). *adjusted for maternal age, maternal education, marrital status, parity, underlying disease, eclampsia, preeclampsia, mode of delivery, gestational age, fetal presentation, birth weight, sex and FCI; facility was adjusted as a random effect; †adjusted for maternal education, matmarital status, parity, underlying disease, preeclampsia, eclampsia, mode of delivery, gestational age, fetal presentation, birth weight and FCI; facility was adjusted as a random effect; ‡adjusted for maternal education, marital status, underlying disease, preeclampsia, eclampsia, mode of delivery, gestational age, fetal presentation, birth weight, sex, corticosteroids and FCI; facility was adjusted as a random effect; §adjusted for maternal age, maternal education, marital status, parity, underlying disease, preeclampsia, eclampsia, mode of delivery, gestational age, fetal presentation, birth weight, sex, corticosteroids and FCI; facility was adjusted as a random effect; ‖adjusted for maternal education, marital status, preeclampsia, eclampsia, mode of delivery, gestational age, fetal presentation, birth weight, sex, corticosteroids and FCI; facility was adjusted as a random effect.

**Region**	**Mode of delivery**	**MICU admission**			**Maternal near miss**			**Maternal death**		
		n/N (%)	Crude OR	(95%CI)	n/N (%)	Crude OR	(95%CI)	n/N (%)	Crude OR	(95%CI)
**Africa**										
GS	VB	11/ 2516 (0.4)	1		NA	NA		7/ 2515 (0.3)	1	
	CS	113/ 473 (23.9)	71.5	(38.1-134.1)	NA	NA		7/ 471 (1.5)	5.4	(1.9-15.5)
MCS	VB	4/ 1860 (0.2)	1		18/ 1860 (1.0)	1		10/ 1860 (0.5)	1	
	CS	11/ 700 (1.6)	7.4	(2.4-23.3)	41/ 700 (5.9)	6.4	(3.6-11.2)	8/ 700 (1.1)	2.1	(0.8-5.4)
**The Americas**										
GS	VB	31/ 2709 (1.1)	1		NA	NA		1/ 2711 (0.04)	1	
	CS	173/ 2453 (7.1)	6.6	(4.5-9.7)	NA	NA		3/ 2455 (0.1)	3.3	(0.3-31.9)
MCS	VB	14/ 1888 (0.7)	1		7/ 1894 (0.4)	1		1/ 1894 (0.1)	1	
	CS	151/ 2370 (6.4)	9.1	(5.3-15.8)	83/ 2382 (3.5)	9.7	(4.5-21.1)	9/ 2382 (0.4)	7.2	(0.9-56.7)
**South East Asia**										
GS	VB	52/ 3687 (1.4)	1		NA	NA		9/ 3687 (0.2)	1	
	CS	72/ 1211 (5.9)	4.4	(3.1-6.4)	NA	NA		2/ 1211 (0.2)	0.7	(0.2-3.1)
MCS	VB	10/ 4197 (0.2)	1		30/ 4197 (0.7)	1		10/ 4197 (0.2)	1	
	CS	48/ 1657 (2.9)	12.5	(6.3-24.8)	41/ 1657 (2.5)	3.5	(2.2-5.7)	3/ 1657 (0.2)	0.8	(0.2-2.8)
**The Western Pacific**										
GS	VB	34/ 1753 (1.9)	1		NA	NA		3/ 1753 (0.2)	1	
	CS	92/ 662 (13.9)	8.2	(5.4-12.2)	NA	NA		2/ 662 (0.3)	1.8	(0.3-10.6)
MCS	VB	14/ 1573 (0.9)	1		6/ 1573 (0.4)	1		2/ 1573 (0.1)	1	
	CS	35/ 790 (4.4)	5.2	(2.8-9.7)	31/ 790 (3.9)	10.7	(4.4-25.7)	1/ 790 (0.1)	1.0	(0.1-11.0)

Table S6. Adverse maternal outcomes by different modes of delivery by regions. NA: Data was not available.

Region	Mode of delivery	APGAR score <7					NICU admission				
		n/N (%)	Crude OR	(95%CI)	aOR	(95%CI)	n/N (%)	Crude OR	(95%CI)	aOR	(95%CI)
**Africa**											
GS	VB	292/ 2473 (11.8)	1		1	*	301/ 2382 (12.6)	1		1	†
	CS	112/ 465 (24.1)	2.4	(1.9-3.0)	1.6	(1.1-2.2)	198/ 437 (45.3)	5.7	(4.6-7.2)	4.8	(2.5-9.0)
MCS	VB	168/ 1671 (10.1)	1		1	‡	459/ 1684 (27.3)	1		1	§
	CS	111/ 640 (17.3)	1.9	(1.5-2.4)	1.8	(1.3-2.6)	295/ 642 (46.0)	2.3	(1.9-2.7)	2.3	(1.7-3.1)
**The Americas**											
GS	VB	323/ 2703 (11.9)	1		1	‖	905/ 2567 (35.3)	1		1	**
	CS	220/ 2453 (9.0)	0.7	(0.6-0.9)	0.6	(0.5-0.8)	1279/ 2398 (53.3)	2.1	(1.9-2.4)	2.4	(2.0-2.8)
MCS	VB	133/ 1778 (7.5)	1		1	*†	577/ 1773 (32.5)	1		1	*‡
	CS	146/ 2327 (6.3)	0.8	(0.7-1.0)	0.8	(0.5-1.1)	1177/ 2325 (50.6)	2.1	(1.9-2.4)	2.1	(1.7-2.6)
**South East Asia**											
GS	VB	485/ 3682 (13.2)	1		1	*§	792/ 3448 (23.0)	1		1	*‖
	CS	118/ 1211 (9.7)	0.7	(0.6-0.9)	0.9	(0.7-1.3)	415/ 1178 (35.2)	1.8	(1.6-2.1)	2.0	(1.6-2.4)
MCS	VB	324/ 3800 (8.5)	1		1	††	1062/ 3821 (27.8)	1		1	†‡
	CS	115/ 1599 (7.2)	0.8	(0.7-1.0)	1.3	(0.9-1.7)	629/ 1608 (39.1)	1.7	(1.5-1.9)	1.6	(1.2-2.1)
**The Western Pacific**											
GS	VB	199/ 1752 (11.4)	1		1	†‡	642/ 1692 (37.9)	1		1	†§
	CS	84/ 662 (12.7)	1.1	(0.9-1.5)	1.1	(0.6-2.0)	356/ 644 (55.3)	2.0	(1.7-2.4)	2.2	(1.4-3.5)
MCS	VB	117/ 1489 (7.9)	1		1	†‖	587/ 1496 (39.2)	1		1	†‖
	CS	51/ 768 (6.6)	0.8	(0.6-1.2)	0.9	(0.6-1.7)	347/ 768 (45.2)	1.3	(1.1-1.5)	1.1	(0.7-1.6)

Table S7. Adverse perinatal outcomes by different modes of delivery by regions. *adjusted for maternal age, maternal education, marital status, parity, underlying disease, eclampsia, mode of delivery, gestational age, fetal presentation, birth weight, and FCI; facility was adjusted as a random effect; †adjusted for maternal age, maternal education, marital status, parity, underlying disease, mode of delivery, gestational age, fetal presentation, birth weight and FCI; facility was adjusted as a random effect; ‡adjusted for maternal age, maternal education, marital status, mode of delivery, gestational age, fetal presentation, birth weight, sex and FCI; facility was adjusted as a random effect; §adjusted for maternal age, maternal education, marital status, eclampsia, preeclampsia, mode of delivery, gestational age, fetal presentation, birth weight, sex and FCI; facility was adjusted as a random effect; ‖adjusted for maternal age, maternal education, marital status, eclampsia, underlying disease, mode of delivery, gestational age, fetal presentation, birth weight, and FCI; facility was adjusted as a random effect; **adjusted for maternal education, eclampsia, preeclampsia, underlying disease, mode of delivery, gestational age, fetal presentation, birth weight, sex, and FCI; facility was adjusted as a random effect; *†adjusted for maternal age, maternal education, marital status, underlying disease, mode of delivery, gestational age, fetal presentation, birth weight, and FCI; facility was adjusted as a random effect; *‡adjusted for maternal age, maternal education, marital status, parity, eclampsia, preeclampsia, underlying disease, mode of delivery, gestational age, fetal presentation, birth weight, sex, and FCI; facility was adjusted as a random effect; *§adjusted for maternal education, eclampsia,

preeclampsia, mode of delivery, gestational age, fetal presentation, birth weight, sex; facility was adjusted as a random effect; *‖ adjusted for maternal age, maternal education, parity, eclampsia, preeclampsia, underlying disease, mode of delivery, gestational age, fetal presentation, birth weight, and sex; facility was adjusted as a random effect; †† adjusted for maternal education, eclampsia, preeclampsia, mode of delivery, gestational age, fetal presentation, birth weight, and sex; facility was adjusted as a random effect;†‡ adjusted for maternal education, parity, eclampsia, preeclampsia, underlying disease, mode of delivery, gestational age, fetal presentation, birth weight, sex, and FCI; facility was adjusted as a random effect;†§ adjusted for maternal education, preeclampsia, mode of delivery, gestational age, birth weight, and FCI; facility was adjusted as a random effect; †‖ adjusted for maternal education, mode of delivery, gestational age, fetal presentation, birth weight, and FCI; facility was adjusted as a random effect.

Region	Mode of delivery	Stillbirth					Early neonatal death					Perinatal death				
		n/N (%)	Crude OR	(95%CI)	aOR	(95%CI)	n/N (%)	Crude OR	(95%CI)	aOR	(95%CI)	n/N (%)	Crude OR	(95%CI)	aOR	(95%CI)
**Africa**																
GS	VB	133/ 2516 (5.3)	1		1	*	82/ 2378 (3.4)	1		1	†	215/2511 (8.6)	1		1	‡
	CS	36/ 473 (7.6)	1.5	(1.0-2.2)	1.0	(0.6-1.6)	20/ 436 (4.6)	1.4	(0.8-2.2)	0.9	(0.4-2.0)	56/ 472 (11.9)	1.4	(1.1-2.0)	0.9	(0.6-1.5)
MCS	VB	175/ 1860 (9.4)	1		1	§	155/ 1683 (9.2)	1		1	‖	330/ 1858 (17.8)	1		1	**
	CS	57/ 700 (8.1)	0.9	(0.6-1.2)	0.6	(0.4-0.8)	76/ 643 (11.8)	1.3	(1.0-1.8)	1.8	(1.1-2.8)	133/ 700 (19.0)	1.1	(0.9-1.4)	0.7	(0.4-1.1)
**The Americas**																
GS	VB	143/ 2713 (5.3)	1		1	*†	129/ 2561 (5.0)	1		1	*‡	272/ 2704 (10.1)	1		1	*§
	CS	56/ 2456 (2.3)	0.4	(0.3-0.6)	0.5	(0.4-0.8)	108/ 2390 (4.5)	0.9	(0.7-1.2)	1.2	(0.8-1.8)	164/ 2446 (6.7)	0.6	(0.5-0.8)	0.8	(0.6-1.1)
MCS	VB	142/ 1894 (7.5)	1		1	*‖	86/ 1751 (4.9)	1		1	††	228/ 1893 (12.0)	1		1	†‡
	CS	63/ 2382 (2.6)	0.3	(0.2-0.5)	0.4	(0.2-0.6)	96/ 2309 (4.2)	0.8	(0.6-1.1)	0.6	(0.4-0.9)	159/ 2372 (6.7)	0.5	(0.4-0.7)	0.9	(0.6-1.5)
**South East Asia**																
GS	VB	239/ 3687 (6.5)	1		1	†§	95/ 3448 (2.8)	1		1	†‖	334/ 3687 (9.1)	1		1	‡‡
	CS	33/ 1211 (2.7)	0.4	(0.3-0.6)	0.5	(0.3-0.7)	39/ 1178 (3.3)	1.2	(0.8-1.8)	1.8	(1.1-2.9)	72/ 1211 (5.9)	0.6	(0.5-0.8)	0.7	(0.5-1.0)
MCS	VB	374/ 4197 (8.9)	1		1	‡§	215/ 3817 (5.6)	1		1	‡‖	589/ 4191 (14.1)	1		1	§§
	CS	48/ 1657 (2.9)	0.3	(0.2-0.4)	0.3	(0.2-0.5)	78/ 1606 (4.9)	0.9	(0.7-1.1)	1.1	(0.8-1.6)	126/ 1654 (7.6)	0.5	(0.4-0.6)	0.6	(0.5-0.8)
**The Western Pacific**																
GS	VB	61/ 1753 (3.5)	1		1	§‖	100/ 1690 (5.9)	1		1	‖‖	161/ 1751 (9.2)	1		1	***
	CS	18/ 662 (2.7)	0.8	(0.5-1.3)	1.1	(0.6-2.1)	43/ 644 (6.7)	1.1	(0.8-1.7)	1.4	(0.8-2.5)	61/ 662 (9.2)	1.0	(0.7-1.4)	0. 9	(0.4-2.0)
MCS	VB	75/ 1573 (4.8)	1		1	‖‖	93/ 1488 (6.2)	1		1	**†	168/ 1563 (10.7)	1		1	**‡
	CS	20/ 790 (2.5)	0.5	(0.3-0.9)	0.5	(0.1-2.2)	37/ 764 (4.8)	0.8	(0.5-1.1)	1.3	(0.7-2.2)	57/ 784 (7.3)	0.7	(0.5-0.9)	1.1	(0.7-1.7)

Table S7. Adverse perinatal outcomes by different modes of delivery by regions (Continue). *adjusted for maternal age, maternal education, parity, underlying disease, eclampsia, mode of delivery, gestational age, fetal presentation, birth weight, and FCI; facility was adjusted as a random effect; †adjusted for maternal education, eclampsia, underlying disease, mode of delivery, gestational age, fetal presentation, birth weight and FCI; facility was adjusted as a random effect; ‡adjusted for maternal age, maternal education, parity, eclampsia, underlying disease, mode of delivery, gestational age, fetal presentation, birth weight, and FCI; facility was adjusted as a random effect; § adjusted for maternal age, maternal education, eclampsia, underlying disease, mode of delivery, gestational age, fetal presentation, birth weight, and FCI; facility was adjusted as a random effect;‖ adjusted for maternal education, marital status, eclampsia, underlying disease, mode of delivery, gestational age, fetal presentation, birth weight, sex and FCI; facility was adjusted as a random effect; **adjusted for maternal age, maternal education, marital status, eclampsia, underlying disease, mode of delivery, gestational age, fetal presentation, birth weight, sex, and FCI; facility was adjusted as a random effect; *†adjusted for maternal age, maternal education, parity, eclampsia, preeclampsia, mode of delivery, gestational age, fetal presentation, birth weight, sex, and FCI; facility was adjusted as a random effect; *‡adjusted for maternal education, marital status, parity, mode of delivery, gestational age, fetal presentation, birth weight, and FCI; facility was adjusted as a random effect; *§adjusted for maternal age, maternal education, marital status, parity, eclampsia, underlying disease, mode of delivery, gestational age, fetal presentation, birth weight, and FCI; facility was adjusted as a random effect; *‖ adjusted for maternal education, underlying disease, mode of delivery, gestational age, fetal presentation, birth weight, sex, and FCI; facility was adjusted as a random effect; †† adjusted for maternal age, maternal education, parity, eclampsia, mode of delivery, gestational age, fetal presentation, birth weight, and FCI; facility was adjusted as a random effect; †‡ adjusted for maternal age, maternal education, underlying disease, mode of delivery, gestational age, fetal presentation, birth weight, sex, and FCI; facility was adjusted as a random effect; †§ adjusted for maternal education, parity, eclampsia, preeclampsia, mode of delivery, gestational age, birth weight, sex, and FCI; facility was adjusted as a random effect; †‖ adjusted for maternal education, eclampsia, preeclampsia, mode of delivery, gestational age, birth weight, and sex; facility was adjusted as a random effect; ‡‡ adjusted for maternal education, parity, eclampsia, preeclampsia, mode of delivery, gestational age, fetal presentation, birth weight, and sex; facility was adjusted as a random effect; ‡§ adjusted for maternal age, maternal education, parity, eclampsia, preeclampsia, underlying disease, mode of delivery, gestational age,

fetal presentation, birth weight, and FCI; facility was adjusted as a random effect; ‡|| adjusted for maternal education, eclampsia, preeclampsia, mode of delivery, gestational age, fetal presentation, birth weight, and FCI; facility was adjusted as a random effect; §§ adjusted for maternal education, parity, eclampsia, preeclampsia, underlying disease, mode of delivery, gestational age, fetal presentation, birth weight, sex, and FCI; facility was adjusted as a random effect; §|| adjusted for maternal education, preeclampsia, mode of delivery, gestational age, fetal presentation, birth weight, and FCI; facility was adjusted as a random effect; |||| adjusted for maternal education, parity, mode of delivery, gestational age, fetal presentation, birth weight, and FCI; facility was adjusted as a random effect; *** adjusted for maternal education, parity, preeclampsia, mode of delivery, gestational age, fetal presentation, birth weight, and FCI; facility was adjusted as a random effect; **† adjusted for maternal age, maternal education, marital status, eclampsia, mode of delivery, gestational age, and birth weight; facility was adjusted as a random effect; **‡ adjusted for maternal education, mode of delivery, gestational age, fetal presentation, birth weight and FCI; facility was adjusted as a random effect.
